# Delegates to the American Medical Association

**Published:** 1857-04

**Authors:** 


*Delegates to the American Medical Association.*

The following are the names of the Delegates appointed by the Illinois State Medical Society to attend the Annual Meeting of the American Medical Association, to be held on the *First* Tuesday in May, in the City of Nashville, Tenn. viz:—Drs. A. H. Luce, of Bloomington; Sam'l Thompson, of Albion; J. V. Z. Blaney, of Chicago; T. R. Edmiston, of Clinton; J. M. Steel, of Grand View; S. T. Trowbridge, of Decatur; S. W. Noble, of Leroy; J. M. Williamson, of Lovington; F. B. Haller, of Vandalia; W. A. Hillis, of Hillsboro; J. N. Cameron, of Charleston; and Thos. Hall, of Toulon.

